# Supplementary material for: Identifying differences in gait adaptability across various speeds using movement synergy analysis
Source: PLoS One. 2021 Jan 7;16(1):e0244582. doi: 10.1371/journal.pone.0244582 (PMC7790368; doi:10.1371/journal.pone.0244582)
Supplement: S1 Data — Both 2D and 3D graphical representations of all validated PMk displayed in Fig 1 of this manuscript are available in video format at the following Github repository: https://github.com/Davidoreilly12/O-Reilly-Federolf-2020. (DOCX) [file pone.0244582.s001.docx]

S1 Data. The data used in this study is publicly available and peer reviewed [35]. Both 2D and 3D graphical representations of all validated PM_k_ displayed in Fig 1 of this manuscript are available in video format at the following Github repository: <https://github.com/Davidoreilly12/O-Reilly-Federolf-2020>.
