# Supplementary material for: Identifying differences in gait adaptability across various speeds using movement synergy analysis
Source: PLoS One. 2021 Jan 7;16(1):e0244582. doi: 10.1371/journal.pone.0244582 (PMC7790368; doi:10.1371/journal.pone.0244582)
Supplement: Attachment — Submitted filename: Response to reviewer.docx [file pone.0244582.s003.docx]

Dear Editor,

I am writing in response to the second minor revision of the manuscript entitled “Identifying differences in gait adaptability across various speeds using movement synergy analysis”. Thank you for the timely turn around in revision. I have read the reviewers comments and you may find the responses to both below:

Reviewer 2

Thank you for your careful consideration of this manuscript and for the quick turnaround in reviewing it. Having read the comments made, you can find a response to both in the following:

**“In lines 119-123 of the introduction, the authors have modified some content to clarify the work that has been done that leads them to conclude the PAk time series represent actions of the neuromuscular system. After reviewing the string of self-citations provided in the manuscript body and the response to reviewers, I cannot conclude whether the field would either agree or disagree with the characterization of PAk as representative of neuromuscular control. I do not wish to stand in the way of good work being published due to a disagreement on the language used to describe a metric. However, it would be my preference that the authors would blend the first and second submission versions of line 121, to keep the more reserved tone and state “…as the extracted acceleration signals are thought to represent the actions of the neuromuscular system on body segments,” as this more adequately reflects the stance of the authors in the comments to the reviewers.”**

In the previously submitted manuscript, the authors made attempts to clarify the definition of the PAk variable noting the current position of the research on muscle synergies and providing a clear description of what this variable is capturing. The authors have also pointed towards previous research using this variable and so wish to stay in line with these studies and their definition used. The authors agree with the amendment recommended by the reviewer and have changed line 121 to the following: “…variables computed from PAk time-series have been of focus in related literature as the extracted acceleration signals are thought to represent the actions of the neuromuscular system on body segments…”. We hope this conveys a more conservative view on the variable to the reader and we again thank the reviewer for their considerations.

**“Regarding the robustness of components across subjects in the methods section lines 163-164, I will acknowledge that I am not used to seeing a cross-validation of this type be the deciding factor in how many principal components get retained. I do still have reservations about maintaining and later drawing conclusions about the PMs that account for such a small proportion of the variance. If the authors are willing to provide the eigenvalues of the PMs displayed in Figure 1, this would be in keeping with what is done in previous studies using this technique (e.g., Haid et al. 2018, Longo et al. 2019). This way the reader will be provided with an additional piece of information that allows them to judge the contribution of these PMs for themselves.”**

The authors agree that eigenvalues should be included. We have therefore created another figure (Fig 2.) directly below Figure 1 that illustrates both the percentage of variance explained by each component and the eigenvalues. The reader can thus see that although a cross validation of these components revealed that these components are adequately stable, some indeed explain a very small amount of the variance in the data.
